# Knockdown of STK39 suppressed cell proliferation, migration, and invasion in hepatocellular carcinoma by repressing the phosphorylation of mitogen-activated protein kinase p38

**DOI:** 10.1080/21655979.2021.1973876

**Published:** 2021-09-14

**Authors:** Jian Chen, Luke Zhou, Jie Yang, Hui Xie, Lin Liu, Youwei Li

**Affiliations:** Department of Hepatobiliary and Pancreatic Surgery, People’s Hospital of DeYang City, Deyang City, Sichuan Province, China

**Keywords:** Hepatocellular carcinoma, stk39, p38, proliferation, invasion

## Abstract

Hepatocellular carcinoma (HCC) is a serious malignant tumor of the liver. It has been reported that serine/threonine kinase 39 (STK39) participates in tumorigenesis. However, the role of STK39 in HCC remains unknown. In this study, the qRT-PCR and western blot assay demonstrated that STK39 expression was enhanced in HCC patients and tissues. Moreover, CCK-8 and colony formation assays confirmed that knockdown of STK39 suppressed SK-HEP-1 and Huh7 cells proliferation. Furthermore, wound healing assay and transwell assay revealed that knockdown of STK39 repressed SK-HEP-1 and Huh7 cells migration and invasion. Interestingly, knockdown of STK39 reduced p-p38/p38 ratio and levels of c-Myc. Consistently, knockdown of STK39 inhibited the HCC tumor growth *in vivo*. In summary, knockdown of STK39 suppressed the proliferation, migration, and invasion of HCC cells by inducing the lower levels of p-p38, which might provide a novel therapeutic target for HCC.

## Introduction

Hepatocellular carcinoma (HCC) is a serious malignant tumor of the liver and has a high rate of mortality. Approximately 80% to 90% of HCC patients are often in the advanced stage of the disease at the time of diagnosis [1]. Therefore, we urgently need to explore the molecular mechanisms of HCC tumorigenesis to develop new clinical therapy for the treatment of HCC. In addition, Serine/threonine kinase 39 (STK39) is connected to the Ste20-like kinase family [2]. Study has suggested that STK39 participated in stress response by activating the expression of p38 MAPK [3]. The expression of STK39 is related to the occurrence of various human diseases, including hypertension, Parkinson’s disease and multiple types of cancer [4]. The levels of STK39 are increasing in diverse types of cancer. Previous study pointed out that the levels of STK39 were promoted during the occurrence and development of osteosarcoma and suppression of STK39 could restrict the proliferation and invasiveness of osteosarcoma cells [5]. The expression of STK39 in breast cancer tissues is also significantly increased, and the inhibition of the expression of STK39 induced the suppression of the proliferation and invasiveness of breast cancer cells [6]. However, whether the levels of STK39 could influence proliferation and invasiveness of HCC cells is unclear.

Furthermore, MAPK pathway plays the crucial role in regulating a variety of responses, such as proliferation, transformation, apoptosis, and stress response of cells [7]. Activation of p38 was the part of the MAPK pathway [8]. The activation of p38 and MAPK pathway could enhance the proliferation and invasiveness of non-small cell lung cancer cells [9]. It has been reported that sauchinone induces apoptosis of Hun-7 cells by activating the JNK/p38 pathway [10]. In addition, hydrazinocurcumin induces apoptosis of HCC cells through activation of p38 MAPK pathway [11], indicating the importance of p38 signaling in the tumorgenesis of HCC. More importantly, knockdown of STK39 restricted the growth and invasion of kidney cancer cells by suppressing p38 and MAPK [12]. However, whether the expression of STK39 could influence the growth and invasiveness of HCC cells remains unclear. In our research, we revealed that the expression of STK39 was promoted in HCC tissues by querying the database (TCGA and Timer). Therefore, we hypothesized that STK39 may influence HCC cell proliferation, migration, and invasion through association with p38 signaling. To confirm this hypothesis, we established STK39-depletion HCC cell lines, and then examined the cell proliferation, migration, and invasion abilities, aiming to explore the mechanism and role of STK39 in HCC.

## Material and methods

**Cell culture and transfection** RPMI culture medium (Hyclone, USA) supplemented with 10% fetal bovine serum (Gibco, USA) was applied for the culture of human normal liver cells (LO2 cells) and HCC cell lines (SK-HEP-1, HCCLM3, and Huh7 cells). Cell lines were placed in 37°C humid atmosphere supplemented with 5% CO_2_. For transfection, the si-NC or si-STK39 (Genechem, Shanghai, China) was transfected into Huh7 and SK-HEP-1 cells with Lipofectamine 2000 (Invitrogen, USA). The transfected cells were incubated for 48 h before experiments.

**Collection of clinical samples** 40 pairs of HCC clinical samples and paired adjacent normal tissues were gathered from the HCC patients. The patients rarely received immune, chemical, or radiation therapy before surgery, and the clinicopathological parameters are summarized in Table 1. These patients were all informed of the trial and approved the performance of these experiments. The experiments were checked and approved by the ethics review committee of People’s Hospital of DeYang city.

**Table 1.** Basic characteristics of patients with liver cancer

clinic copathological varialble	STK39 expression		
	Low(n=20)	High(n=20)	P
Age			
<50	8	6	0.507
≥50	12	14	
Gender			
male	7	6	0.736
female	13	14	
AFP			
400ng/mL	5	4	0.705
≥400ng/mL	15	16	
Tumor size			
<5cm	14	5	0.004
≥5cm	6	15	
Cirrhosis			
negative	2	4	0.661
positive	18	16	
Metasis			
negative	19	13	0.044
positive	1	7	
TNM			
I-II	17	10	0.018
III-IV	3	10	

**Animal assays** 10 nude male mice were obtained from the Shanghai Institute of Zoology (CAS, Shanghai, China). Then, these mice were divided into two groups (five mice per group). Next, 5 × 10^6^ SK-HEP-1 cells were implanted into these mice (sh-NC and sh-STK39 groups) by the subcutaneous injection. After three weeks, these mice were sacrificed with the euthanasia and the tumor tissues were collected for the research. The volume and weight of the tumor tissues were measured after the sacrifice of these mice. The animal assays were also checked and approved by the ethics review committee of People’s Hospital of DeYang city.

**Immunohistochemistry** The tumor tissue was embedded in paraffin and cut into slices of 3–5 mm thickness. Next, these slices were dewaxing and the BSA (Beyotime, China) was applied for the block of these slices. After that, primary antibodies were incubated with tissues at 4°C overnight. STK39 (Abcam, ab128894), Ki-67 (Abcam, ab16667) and MMP-2 (Abcam, ab86607) were applied for this research. Next, secondary antibodies (goat anti-rabbit IgG, Abcam, ab172730 and goat anti-mouse IgG, Abcam, ab150113) were incubated with these tissues. Finally, these tissues were hatched with the TMB substrate (Millipore, USA) and observed under the pathological microscope (Leica, Germany). The IHC was quantified to detect target protein expression levels according to the following intensity score criteria: 0, negative staining; 1, weak staining; 2, moderate staining; and 3, strong staining. The percentage of positive score: percentage of positive <5%, 0; 5–25%, 1; 25–50%, 2; 50–75%, 3; and >75%, 4. The final scores were calculated as the score of positive expression percentage multiplied by the intensity score.

**Cell counting kit-8 (CCK-8) assays** The cell viability was determined by CCK-8 (Dojindo, Japan) assay. When the cells cultured in 96-well plate grew at a density of 1 × 10^4^/mL, the CCK-8 solution was added to each well at 24, 48 and 72 hours. Then, the absorbance of OD_450nm_ was measured by spectrophotometer (Thermo Fisher Scientific, USA).

**Clone formation assays** The cells were plated into the 60 mm culture dish (200 cells per dish). Then, the cells were cultured in the incubator for 2 weeks. After that, the cells were fixed with the 70% ethanol solution. Crystal violet solution (Invitrogen, USA) was applied for the cell staining. Number of clones was calculated under the inverted phase contrast microscope (Olympus, Japan).

**Wound healing assays** Six well plates were used for the culture of the cells. Before the performance of these experiments, the cells were incubated with the serum-free medium for 12 hours. After that, the tweezers were used for the creation of scratch on these cells. Then, the scratch was photographed with the microscope (Olympus, Japan). After 24 hours, the scratch was photographed again. The width of the scratch at 0 h and 24 h was measured with the Image J (NIH, USA).

**Transwell** Before performance of this experiment, cells were cultured with serum-free medium for 12 hours. At the same time, the serum-free medium was used for the attenuation of matrigel (BD, USA). Next, the matrigel was injected in the upper chamber (8 μm, Corning, USA). And the lower chamber was filled with the complete medium. Thereafter, the cells were added into the upper chamber and incubated in the incubator (24 hours). Then, cells on the reverse of the bottom membrane were dyed with the crystal violet solution (Invitorgen, USA). Finally, the cells were photographed and observed under the microscope (Olympus, Japan).

**Real-time polymerase chain reaction (RT-PCR)** RNA was extracted by trizol (Takara, USA) methods. Then, commercial kits (Takara, Japan) were used for the reverse transcription of the RNA. After that, the cDNA was mixed with the SYBE Green (Thermo Fisher Scientific, USA) and amplified with the ABI7500 system (Thermo Fisher Scientific, USA). Results of PCR were analyzed with the 2^−∆∆Ct^ method. The primer sequences were listed as following: STK39 (ENSG00000198648) forward primer 5ʹ-TCTGCTGGCTTGGTGGATG-3ʹ reverse primer 5ʹ-AGGGAGGGTTGAAG GGAGTAG-3ʹ GAPDH (ENSG00000111640) forward primer 5ʹ-CGGAGTCAACGGATTTGGTCGTAT-3ʹ reverse primer 5ʹ-AGCCTTCTCCATGGTGGTGAAGAC-3ʹ were the primers applied for this research.

**Western blotting** Protein samples were extracted by the lysis buffer (Beyotime, China). Then, concentration of these samples was measured with the BCA methods. After that, the proteins were separated with the 10% SDS-PAGE gel (Beyotime, China). Then, proteins were transferred onto PVDF membranes (Millipore, USA). Membranes were blocked with BSA (Invitrogen, China). Primary antibodies were incubated with these membranes at 4°C overnight, Including anti-STK39 (Abcam, ab106936), p21 (Abcam, ab109520), PCNA (Abcam, ab92552), MMP-2 (Abcam, ab92536), MMP-9 (Abcam, ab76003), p-p38 (Abcam, ab178867), p38 (Abcam, ab170099) c-Myc (Abcam, ab32072) and GAPDH (Abcam, ab8245) antibodies. The primary antibodies (1:1000) were attenuated with BSA (Invitrogen, USA). Next, membranes were hatched with the secondary antibodies (Goat anti-rabbit IgG, Abcam, ab150077 and Goat anti-mouse IgG, Abcam, ab150113) for 2 hours. Substrate (Millipore, USA) was used for the development of bands. The quantification of these bands was performed with the Image J (NIH, USA).

**Statistical analysis** Graphpad Prism 6.0 was applied for the analysis of the data in this research. The data in this research was displayed as mean±SD. All the assays of this study were repeated for three times. Student’s *t* test was applied for the comparison between diverse groups. The difference was considered as statistical significance until the values of *p* was less than 0.05.

## Results

To explore the role of SKT39 in HCC cells, the Huh7 and SK-HEP-1 cells were transfected with si-NC or si-STK39. Thereafter, the cell migration, invasion, and proliferation were detected, and the results suggested that knockdown of SKT39 suppressed HCC cells proliferation, migration, and invasion. Interestingly, depletion of SKT39 repressed the phosphorylation of p38. Consistently, the *in vivo* experiment demonstrated that knockdown of SKT39 inhibited tumor growth in mice. Thus, we concluded that knockdown of STK39 suppressed cell proliferation, migration, and invasion of HCC by repressing the phosphorylation of p38.

**Figure 1.**
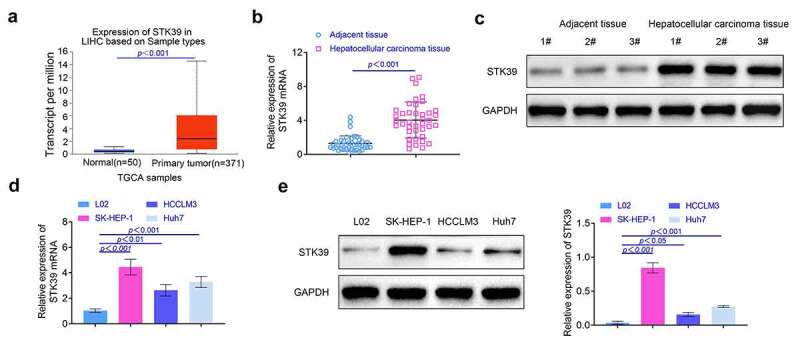
**The expression of STK39 was promoted in HCC tissues**. (a) The expression of STK39 in HCC tissues was explored with the database. (b) The expression of STK39 in HCC tissues was determined with the RT-PCR, n = 40. (c) The levels of STK39 in HCC tissues was detected with the western blotting. (d, e) The expression of STK39 in HCC cells was detected with the RT-PCR and western blotting, respectively. Each experiment repeated for at least three times. **p < 0.01, ***p < 0.001 vs. L02 cells

**The expression of STK39 was enhanced in the HCC tissues** To detect the effect of STK39 on the development of HCC, we examined the levels of STK39 in HCC tissues and pericarcinomatous tissues. The data from TCGA dataset showed that the HCC patients have the higher levels of STK39 in HCC tissues compared with the normal people (*p* < 0.001, Figure 1a). Next, we collected the clinical samples and detected levels of STK39 in these tissues. Results (Figure 1b and Figure 1c) indicated that the levels of STK39 in HCC tissues were higher than that in pericarcinomatous tissues (*p* < 0.01). After that, we also explored the levels of STK39 in human normal liver cells (LO2 cells) and HCC cells (SK-HEP-1, HCCLM3, and Huh7 cells). As shown in Figure 1d and Figure 1e, the expression of STK39 was enhanced in HCC cells compared with the human normal liver cells (*p* < 0.01). The results of this part suggested that levels of STK39 was promoted in HCC tissues.

**Figure 2.**
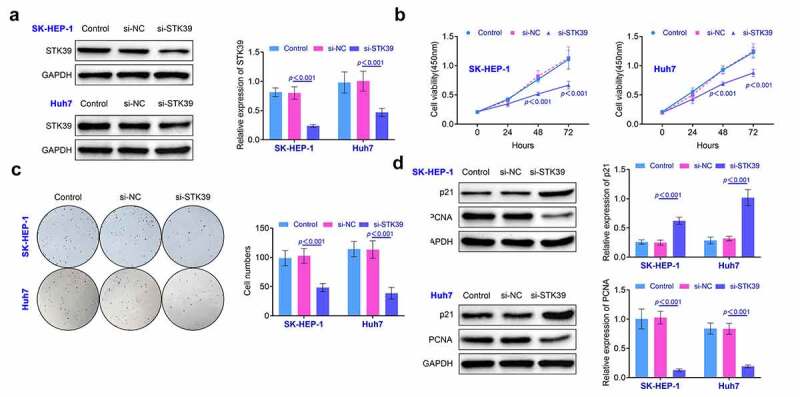
**Knockdown of STK39 repressed the proliferation of HCC cells**. (a) The expression of STK39 in HCC cells was determined with the western blotting. (b) Cell viability of HCC cells was detected with the CCK-8 assays. (c) The proliferation of HCC cells was determined with the clone formation assays. (d) The expression of p21 and PCNA in HCC cells was detected with the western blotting. Each experiment repeated for at least three times. **p < 0.01 vs. si-NC

**Knockdown of STK39 repressed the growth of HCC cells** SK-HEP-1 and Huh7 cells were selected for the establishment of the STK39 knockdown HCC cells. According to the results (Figure 2a), the levels of STK39 was suppressed in cells after the transfection (*p* < 0.01). Results (Figure 2b) of CCK-8 assay showed that the cell viability was significantly decreased at 24, 48 and 72 hours after knockdown of STK39 (*p* < 0.01). Similarly, the results (Figure 2c) of colony formation assays also showed that knockdown of STK39 suppressed the formation of colony of SK-HEP-1 and Huh7 cells (*p* < 0.01). Moreover, the expression of proliferation-related proteins (p21 and PCNA) was detected by western blotting. As shown in Figure 2d, the expression of p21 was promoted while the levels of PCNA were decreased after the knockdown of STK39 (*p* < 0.01). These results indicated that the knockdown of STK39 repressed the proliferation of HCC cells.

**Figure 3.**
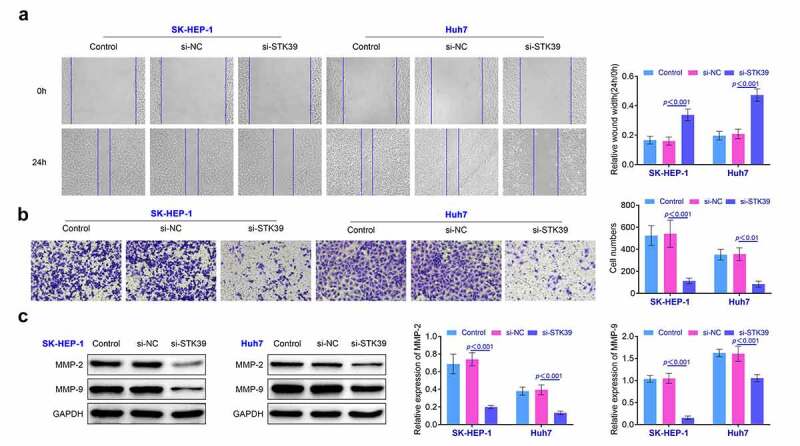
**Knockdown of STK39 inhibited the invasiveness of HCC cells**. (a) The migration of HCC cells was detected with the wound healing assays. (b) Transwell assays were used for the detection of the invasion of HCC cells. (c) The expression of MMP-2 and MMP-9 in HCC cells was detected with the western blotting. Each experiment repeated for three times. **p < 0.01 vs. si-NC

**Knockdown of STK39 restricted the migration and invasiveness of HCC cells** In this part, wound healing assays were performed to explore the changes of the migration of STK39 knockdown HCC cells. Results (Figure 3a) suggested that repression of STK39 restricted the migration of SK-HEP-1 and Huh7 cells (*p* < 0.01). Moreover, results (Figure 3b) of transwell also revealed that the invasiveness of SK-HEP-1 and Huh7 cells was suppressed after the repression of STK39 (*p* < 0.01). The detection of MMP-2 and MMP-9 also revealed that the knockdown of STK39 suppressed the expression of MMP-2 and MMP-9 in SK-HEP-1 and Huh7 cells (*p* < 0.01, Figure 3c). These results implied that the knockdown of STK39 repressed the invasiveness of HCC cells.

**Figure 4.**
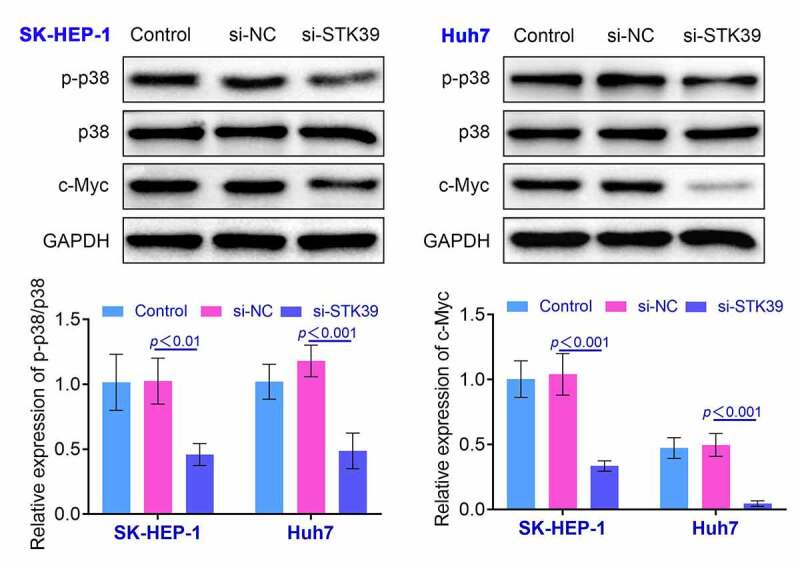
**Knockdown of STK39 induced the decreasing of the ratio of p-p38/p38**. The expression of p-p38, p38 and c-Myc in HCC cells was determined with the western blotting. Each experiment repeated for three times. **p < 0.01 vs. si-NC

**Knockdown of STK39 inhibited the expression of p-p38 in HCC cells** Previous research indicated that STK39 could regulate p38-related pathway and affect the development of renal carcinoma [12]. Therefore, we detected the levels of p-p38 and c-Myc by the western blotting. We found that the ratio of p-p38/p38 was down-regulated and the expression of c-Myc was suppressed in SK-HEP-1 and Huh7 cells after the knockdown of STK39, indicating that knockdown of STK39 deactivated p38 signaling (*p* < 0.01, Figure 4). These results also implied that restriction of STK39 affected the growth and invasiveness of HCC cells by modulating the expression of p-p38.

**Figure 5.**
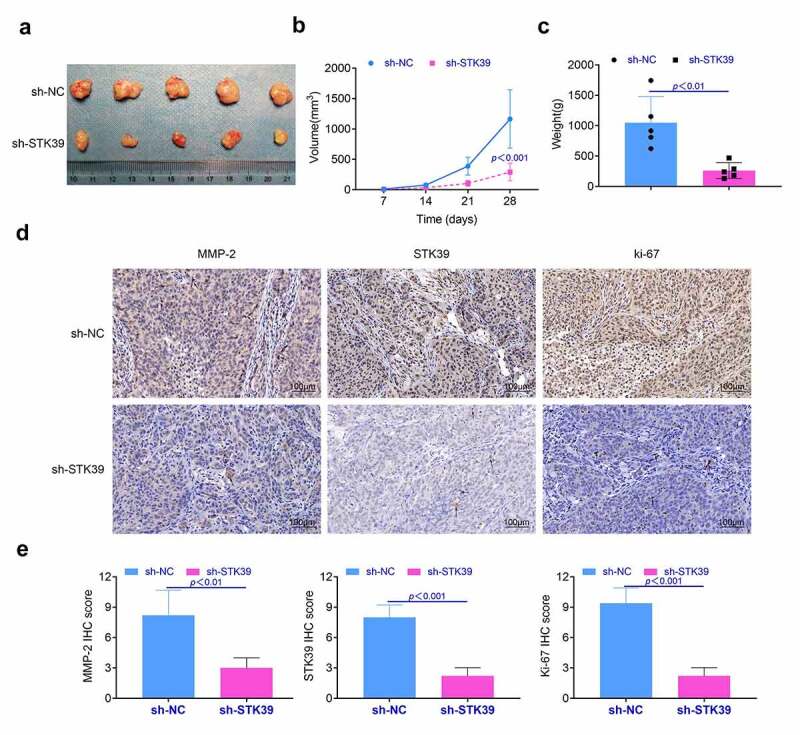
**Knockdown of STK39 suppressed the proliferation of HCC cells *in vivo***. (a) The proliferation of HCC cells in vivo was detected with the animal assays. (b, c) Volume and weight of tumor tissues were measured after the sacrifice of these mice. (d) The expression of Ki-67, MMP-2 and STK39 in tumor tissues was determined with the immunohistochemistry, the IHC scores were calculated. Five mice per group. Each experiment repeated for three times. **p < 0.01 vs. sh-NC

**Knockdown of STK39 suppressed the proliferation of HCC tumor *in vivo*** In this part, we detected the effect of STK39 on the formation of HCC tumor *in vivo*. Results (Figure 5a) showed that the knockdown of STK39 restricted the growth of HCC tumor *in vivo*. The tumor volume and weight of STK39 knockdown group was lower than those of negative control group (*p* < 0.01, Figure 5b and Figure 5c). Furthermore, results (Figure 5d) of immunohistochemistry showed that the expression levels of Ki-67, MMP-2 and STK39 in tumor of STK39 knockdown group were lower than that in negative control group (*p* < 0.01). These results suggested that knockdown of STK39 suppressed the proliferation and invasion of HCC cells *in vivo*.

## Discussion

HCC is a malignant tumor with extremely high mortality and the main cause of the cancer-induced death [13]. Furthermore, the incidence of this disease has been rising in Asia and Africa in recent years [14]. At present, surgical removal of tumors is still the main clinical treatment option for HCC. However, due to the high recurrence rate, the postoperative survival period of patients is still short [15]. Although the diagnosis and treatment technology of liver cancer has made significant progress, the survival rate of liver cancer patients has not significantly improved [16]. The main reason for the recurrence of HCC is the strong proliferation and metastasis of HCC cells [17]. The metastasis of tumor cells is a complex biological process, which is often related to the changes of the expression of multiple genes in cells [18]. And the changes in the expression of vascular endothelial growth factor (VEGF) and transforming growth factor-β ([5] transforming growth factor-β, TGF-β) also played a critical role in the proliferation and metastasis of HCC cells [19]. In addition, STK39 maintained the stability of blood pressure by regulating ion homeostasis in mammals [20]. Previous study showed that the expression of STK39 was associated with the development of multiple types of cancer [21]. And the expression of STK39 is also related to the malignant degree of some cancers [22,23]. Overexpression of STK39 could strengthen the proliferation and invasiveness of osteosarcoma cells [5]. Moreover, knockdown of STK39 suppressed the proliferation and invasion of non-small cell lung cancer cells by inducing the cell cycle arrest and apoptosis of these cells [22]. In this study, we revealed that the expression of STK39 was increased in HCC tissues and cells. Suppression of STK39 restricted the proliferation and invasiveness of HCC cells. Moreover, lower levels of STK39 also suppressed the proliferation of HCC tumor *in vivo*. These results indicated that STK39 is the promoting factor of the occurrence and development of HCC and the STK39 has the potential to be explored as a therapeutic target of HCC. It is remarkable that SP1-mediated STK39 up-regulation leads to the increased proliferation, migration, invasion, and EMT of HCC cells via activating TGF-β1/Smad2/3 pathway, which is highly consistent with our results [24].

Furthermore, p38/MAPK pathway was associated with the occurrence and development of the inflammation of diverse tissues [25]. Some studies revealed that the activation of p-p38 was related to the proliferation of multiple types of cancer cells [26]. Another research suggested that the PP2A could suppress the proliferation and invasiveness of cervical cancer cells by inducing the dephosphorylation of p-p38 [27]. These results implied that the phosphorylation of p38 has the potential to promote the proliferation, migration, and invasion of cervical cancer cells. Activation of p38 could also promote the proliferation and invasion of prostate cancer cells [28]. Moreover, the study also revealed that the knockdown of STK39 suppressed the proliferation and invasion of renal carcinoma cells by restricting the phosphorylation of p38 [12]. In our study, we found that the knockdown of STK39 induced the decrease in the ratio of p-p38/p38 and repressed the expression of c-Myc in HCC cells. These results suggested that knockdown of STK39 suppressed the proliferation and invasiveness of HCC cells by restricting the phosphorylation of p38.

## Limitation

There are still limitations in our results. First, the HCC tissues were collected from 40 pairs of HCC tissues and adjacent normal tissues, indicating that our clinical results were based on limited samples and have to be validated by larger samples. Second, it is conceivable that knockdown of STK39 suppressed the proliferation and invasiveness of hepatocellular carcinoma cells by repressing the phosphorylation of p38. Nevertheless, SKT39 may involve additional signaling pathways, which needs to be identified in further studies.

## Conclusion

In summary, knockdown of STK39 suppressed HCC cells proliferation, migration, and invasion via inhibiting the phosphorylation of p38, providing a novel potential therapeutic target for HCC.

## Highlights

The expression of STK39 was promoted in HCC tissues.

Knockdown of STK39 repressed the proliferation of HCC cells.

Knockdown of STK39 repressed the invasiveness of HCC cells.

Knockdown of STK39 affected the proliferation and invasion of HCC cells by modulating the expression of p-p38.

Knockdown of STK39 suppressed the proliferation and invasion of HCC cells *in vivo*.
